# A comparative study on the *in vivo* degradation of poly(L-lactide) based composite implants for bone fracture fixation

**DOI:** 10.1038/srep20770

**Published:** 2016-02-09

**Authors:** Zongliang Wang, Yu Wang, Yoshihiro Ito, Peibiao Zhang, Xuesi Chen

**Affiliations:** 1 Key Laboratory of Polymer Ecomaterials, Changchun Institute of Applied Chemistry, Chinese Academy of Sciences, Changchun 130022, PR China; 2 Nano Medical Engineering Laboratory, RIKEN, 2-1 Hirosawa, Wako, Saitama 351-0198 Japan; 3 Emergent Bioengineering Materials Research Team, RIKEN Center for Emergent Matter Science, 2-1 Hirosawa, Wako, Saitama 351-0198 Japan; 4 University of Chinese Academy of Sciences, Beijing 100039, PR China

## Abstract

Composite of nano-hydroxyapatite (n-HAP) surface grafted with poly(L-lactide) (PLLA) (g-HAP) showed improved interface compatibility and mechanical property for bone fracture fixation. In this paper, *in vivo* degradation of n-HAP/PLLA and g-HAP/PLLA composite implants was investigated. The mechanical properties, molecular weight, thermal properties as well as crystallinity of the implants were measured. The bending strength of the n- and g-HAP/PLLA composites showed a marked reduction from an initial value of 102 and 114 MPa to 33 and 24 MPa at 36 weeks, respectively. While the bending strength of PLLA was maintained at 80 MPa at 36 weeks compared with initial value of 107 MPa. The impact strength increased over time especially for the composites. Significant differences in the molecular weight were seen among all the materials and g-HAP/PLLA appeared the fastest rate of decrease than others. Environmental scanning electron microscope (ESEM) results demonstrated that an apparently porous morphology full of pores and hollows were formed in the composites. The results indicated that the *in vivo* degradation of PLLA could be accelerated by the g-HAP nanoparticles. It implied that g-HAP/PLLA composites might be a candidate for human non-load bearing bone fracture fixation which needs high initial strength and fast degradation rate.

Hydroxyapatite (HAP) and poly(L-lactic acid) (PLLA) composites have been widely studied as biodegradable materials in clinical applications, such as bone fracture fixations, suture anchors, craniomaxillofacial fixation, interference screws, and meniscus repair^1^. Using HAP/PLLA as bone fracture fixation materials can not only avoid removing the devices with a second operation, but also preventing the stress-shielding atrophy and weakening the fixed bone as the metal fixation devices did^2^,^3^. However, some significant disadvantages are required to be improved, including the interface bonding ability between the two phases, mechanical properties and the *in vivo* degradation behavior^4^,^5^. Even if nano-hydroxyapatite (n-HAP) was the inorganic component of bone and has good osteoinductivity and biocompatibility, n-HAP particles were in lack of adhesion with the PLLA matrix in HAP/PLLA composite. In order to improve the interfacial adhesion between the HAP particles and the PLLA matrix, in our previous study, the PLLA based nanocomposite of surface grafted HAP with ring-opening polymerization of L-latide (LLA) (g-HAP) was prepared^6^. The PLLA molecules grafted on the HAP surfaces, as inter-tying molecules, played an important role in improving the adhesive strength between the particles and the polymer matrix. The results indicated that PLLA could be strengthened as well as toughened by g-HAP nanoparticles. However, the influence of g-HAP incorporation on the *in vivo* degradation of g-HAP/PLLA composite need further investigated.

It is very important to investigate the degradation behavior of biodegradable materials as degradation rate is a critical factor affecting bone fracture healing. Several studies have focused on the *in vitro* and *in vivo* degradation of PLLA based composites. However, there are conflicting results on the effect of bioceramics filler on resorption rates. Some researchers reported that addition of nano filler slowed down the degradation of composite. For example, Bleach *et al.*^7^ found that unfilled PLLA absorbed more water and showed greater mass loss than the samples containing hydroxyapatite (HA) or tricalcium phosphate (TCP) fillers after immersing in simulated body fluid (SBF) for 12 weeks. Niemelä *et al.*^8^ reported that the degradation of the β-TCP/PLA composite was slower than that of PLA. Araújo *et al.*^9^ observed that clay mineral incorporation in PLA matrix enhanced the polymer thermal stability.

Whereas other authors have observed increase of the degradation rate in the presence of HA, TCP or other fillers, attributed to the particle/matrix interface and the hydrophilicity of the fillers. Delabarde *et al.*^10^ and Jiang *et al.*^4^ reported that incorporation of HA into HA/PLA (or HA/PLGA) composites could accelerate degradation at the matrix/particle interfaces. Addition of β-TCP^11^ and soluble calcium phosphate (CaP) glass^12^ were also found to accelerate the degradation of the PLA. Besides, montmorillonite, nanoclay and titanium dioxide (TiO_2_) nanoparticles were also proved to decrease the thermal stability and accelerate the *in vitro* degradation of PLA matrix^13^,^14^,^15^,^16^. In addition to the above *in vitro* studies, Furukawa *et al.*^5^ evaluated the *in vivo* degradation of PLA based composite rods and found that addition of HA showed a faster rate of degradation.

As a novel modification method, n-HAP surface grafted of PLLA (g-HAP) attracted researchers’ attentions and Li *et al.*^17^ found that the g-HAP particle slowed down the thermal degradation of PLA polymer matrix. Based on our previous study, in the present work, we tried to focus our research on the comparative *in vivo* degradation study of g-HAP/PLLA and n-HAP/PLLA composites.

## Results

### Mechanical properties

**Figure 1:**
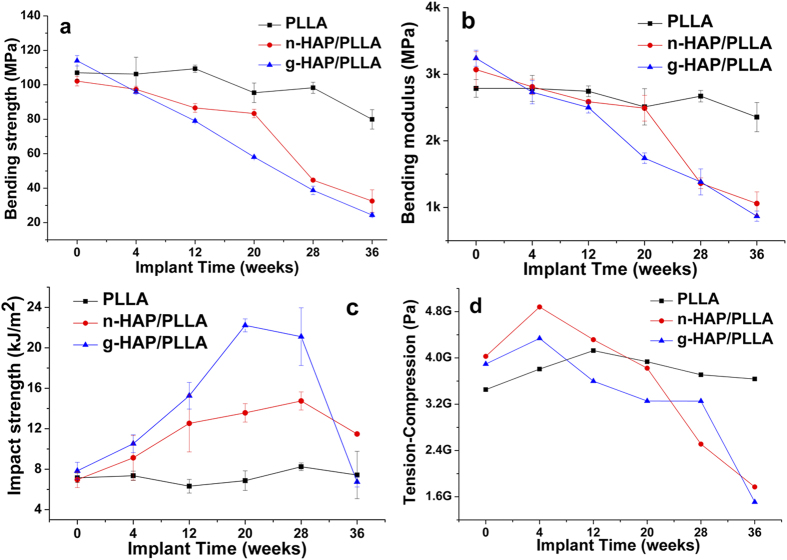
Changes in the bending strength (**a**), bending modulus (**b**), impact strength (**c**) and torsion test (**d**) of PLLA, n- and g-HAP/PLLA at 0-36 weeks post-surgery.

The mechanical property changes of the implants over time after surgery were shown in Fig. 1. The initial bending strength of g-HAP/PLLA composites (114 ± 3 MPa) was a little higher than that of PLLA (107 ± 4 MPa). While the initial bending strength of n-HAP/PLLA composites (102 ± 3 MPa) was slightly lower than that of PLLA. The bending strength of the n- and g-HAP/PLLA composites decreased gradually after surgery according to Fig. 1a. There was a slight decrease of n-HAP/PLLA composites 20 weeks after surgery, subsequently decreased remarkably. They maintained 81.6% of their initial values at 20 weeks and 43.8% at 28 weeks. The bending strength of g-HAP/PLLA composites decreased constantly post-surgery and maintained 51.0% of their initial values at 20 weeks and 34.0% at 28 weeks. At 36 weeks the g-HAP/PLLA composites maintained only 21.4% of their initial bending strength, while the n-HAP/PLLA composites maintained 31.8%. On the contrary, there was only a little reduction of PLLA compared with the two composites and maintained 74.7% of initial bending strength even at 36 weeks. There was a significant difference among the three materials which was listed in Table 1.

The bending modulus retention of the materials were similar with the bending strength retention as shown in Fig. 1b. The n-HAP/PLLA composites maintained 81.1% of their initial values at 20 weeks and 44.4% at 28 weeks. The bending modulus of g-HAP/PLLA composites maintained 53.7% of their initial values at 20 weeks and 42.7% at 28 weeks. At 36 weeks the g-HAP/PLLA composites maintained only 26.8% of their initial bending modulus, while the n-HAP/PLLA composites maintained 34.5%. Correspondingly, PLLA maintained 81.6% of initial bending modulus even at 36 weeks. Details of statistical analysis were shown in Table 2.

Interestingly, the impact strength exhibited completely difference from the bending strength and modulus as *in vivo* degradation (Fig. 1c). There was a slight increase in the impact strength of n- and g-HAP/PLLA composites 4 weeks and 12 weeks after surgery. Unlike n-HAP/PLLA composites with a slight increase to 195% and 211.9% of their initial impact strength at 20 and 28 weeks, it increased remarkably to 283.5% and 269.3% of initial impact strength for g-HAP/PLLA composites. Moreover, the impact strength of g-HAP/PLLA were always higher than that of n-HAP/PLLA composites at any time interval prior to 28 weeks. The impact strength decreased at 36 weeks for all the two composites, especially the g-HAP/PLLA composites. Conversely, there was no obvious change of PLLA compared with the two composites and maintained 103.8% of initial impact strength even at 36 weeks. Details of statistical analysis were shown in Table 3.

As shown in Fig. 1d, the viscoelasticity of PLLA, n- and g-HAP/PLLA composites were evaluated at 37 °C. The viscoelasticity slightly increased at 4 weeks and then decreased gradually to 43.9% and 38.8% of their initial values at 36 weeks for n- and g-HAP/PLLA composites. However, there was no obvious change of PLLA with 105.5% of their initial values at 36 weeks.

### Molecular weight change

**Figure 2:**
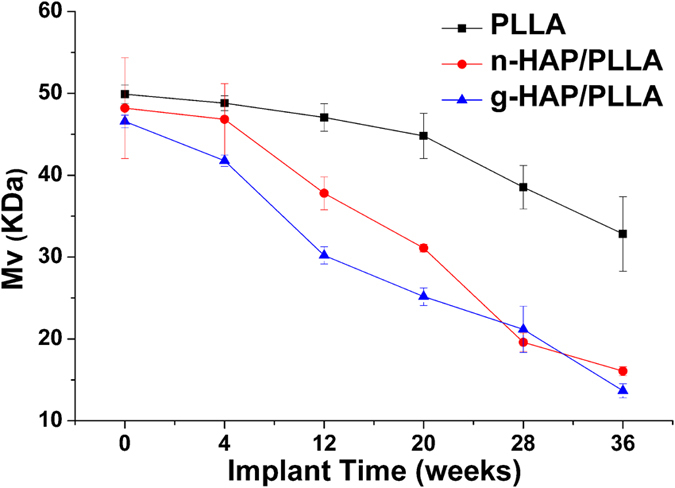
Changes in viscosity-average molecular weight (*M*v) of PLLA, n- and g-HAP/PLLA at 0–36 weeks post-surgery.

Fig. 2 showed changes in molecular weight for PLLA in all the implants at all the time intervals. The molecular weight of the g-HAP/PLLA composites at 4, 12, 20, 28 and 36 weeks after implantation were 89.7, 64.8, 54.1, 45.5 and 29.4% of their initial values, respectively. While those of the n-HAP/PLLA composites were 97.1, 78.4, 64.5, 40.7 and 33.4%, respectively. Conversely, the molecular weights of PLLA at 4, 12, 20, 28 and 36 weeks after implantation were 97.8, 94.4, 89.8, 77.2 and 65.7% of their initial values. Thus, the g-HAP/PLLA composites exhibited a significantly greater decrease in molecular weight than the n-HA/PLLA composites and the composites decreased at a significantly faster rate than the unfilled PLLA samples.

### Thermal and crystalline properties

**Figure 3:**
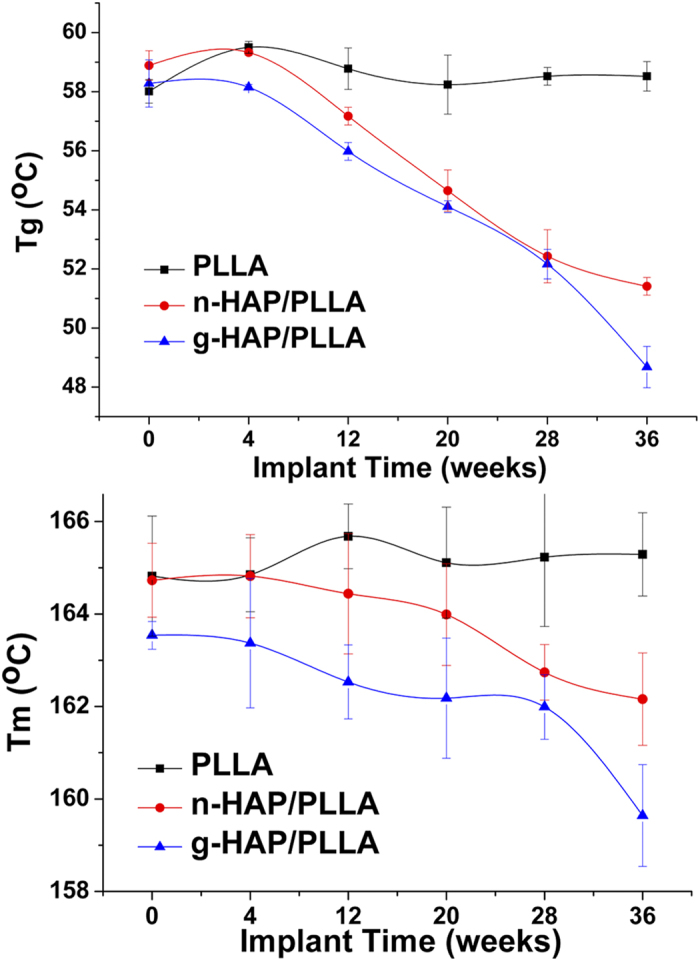
Changes in Tg and Tm of PLLA, n- and g-HAP/PLLA at 0–36 weeks post-surgery.

The thermal and crystalline properties of the samples before and throughout *in vivo* degradation period were shown in Fig. 3 and Table 4. The glass transition (*T*g) and melting temperature (*T*m) of PLLA matrix for both the composites were observed to decrease and consequently the crystallinity were found to increase with *in vivo* degradation. Before implantation, the initial *T*g and *T*m of n- and g-HAP/PLLA composites were seen to be around 58.9, 164.7 °C and 58.3, 163. °C, respectively. A significant decrease in *T*g and *T*m of n- and g-HAP/PLLA composites by approximately 7.5, 2.5 °C and 9.6, 3.9 °C, respectively, were observed after *in vivo* degradation for 36 weeks. However, there was almost no change in *T*g and *T*m of pure PLLA samples.

As shown in Table 4, the initial values of the crystallinity of n- and g-HAP/PLLA composites were a little higher than that of pure PLLA. Both the composites showed similar patterns of increasing crystallinity until 20 weeks after implantation and pure PLLA showed increasing crystallinity until 28 weeks. However, the g-HAP/PLLA composites exhibited the highest values among all the materials and pure PLLA always showed the lowest values in crystallinity at any time interval.

### Surface and fracture ESEM morphology

**Figure 4:**
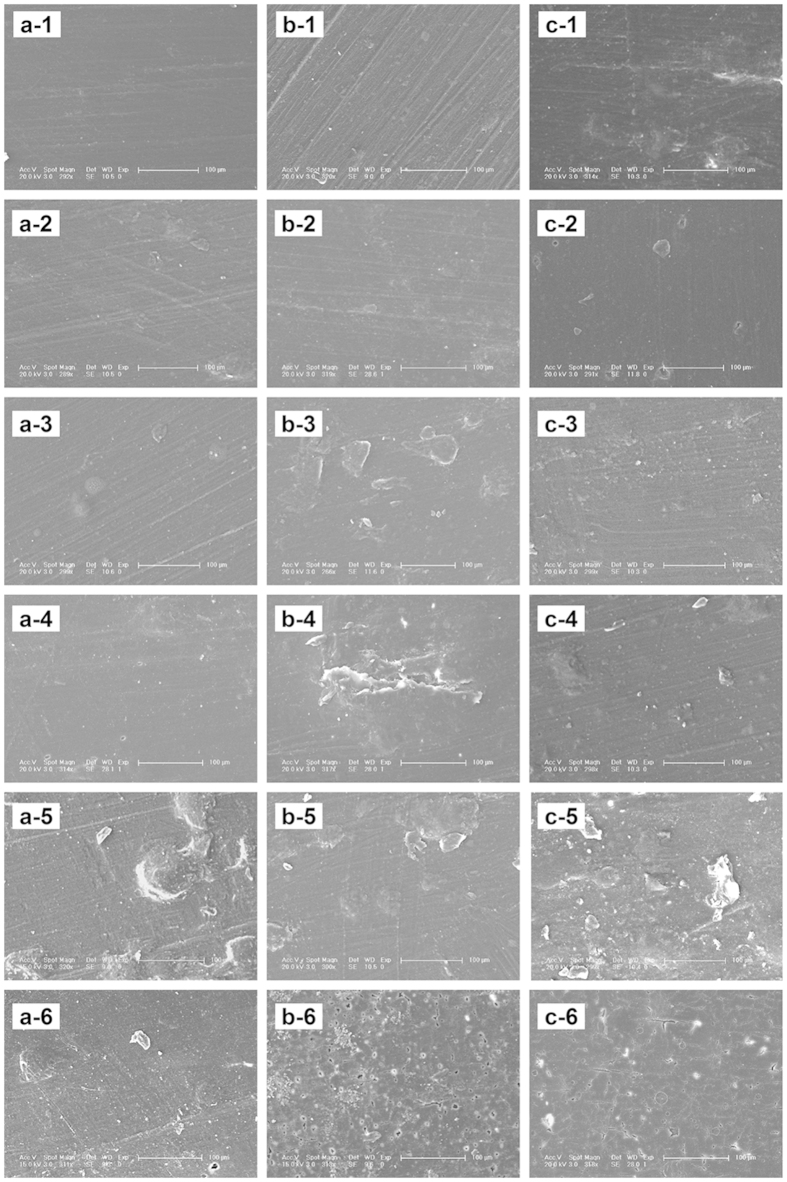
Surface ESEM micrographs of the intramuscular implants of PLLA (**a**), n-HAP/PLLA (**b**) and g-HAP/PLLA (**c**) at 0(−1), 4(−2), 12(−3), 20(−4), 28(−5) and 36(−6) weeks post-surgery.

No apparent macroscopic changes were observed in the surface of the materials removed from the surrounding tissues over time after implantation. The surface ESEM morphology of PLLA, n- and g-HAP/PLLA composites at different time interval before and after implantation was shown in Fig. 4. More surface roughness was noted on the surface of all materials over time and some small pores appeared on the materials at 36 weeks, especially for the composites.

**Figure 5:**
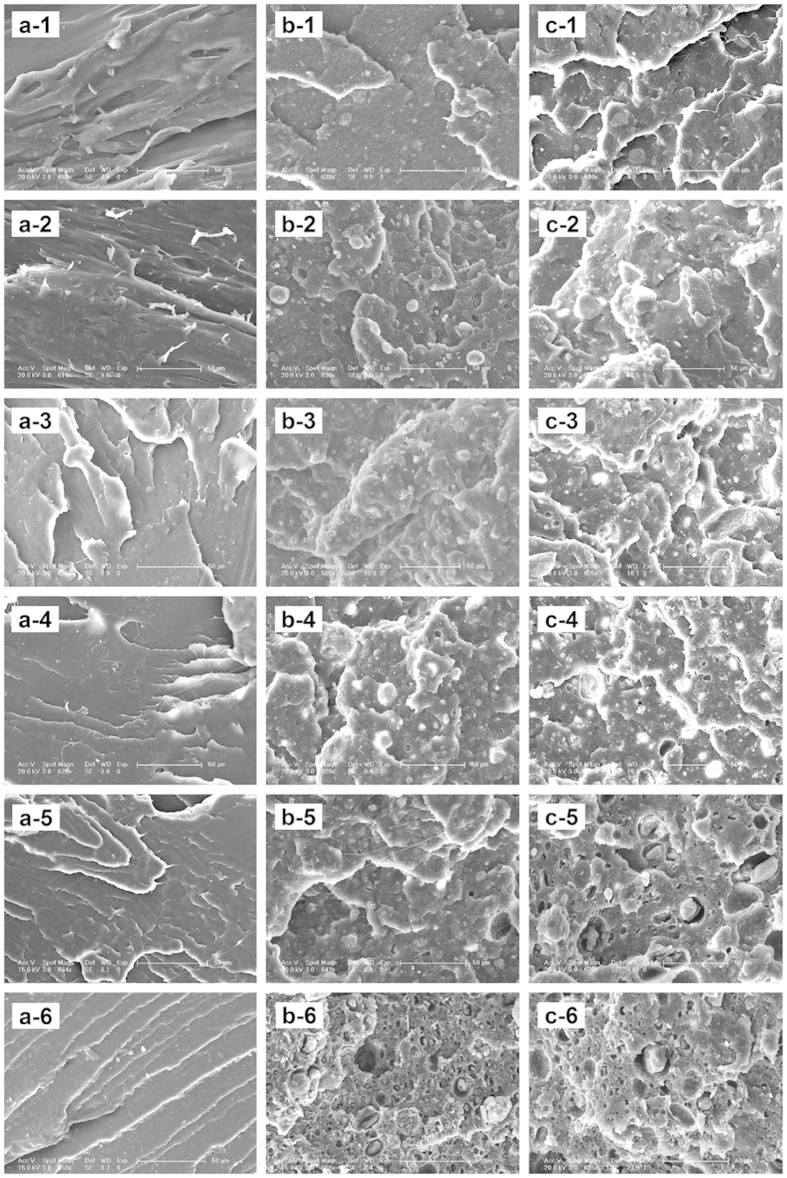
Fracture ESEM micrographs of the intramuscular implants of PLLA (**a**), n-HAP/PLLA (**b**) and g-HAP/PLLA (c) at 0(−1), 4(−2), 12(−3), 20(−4), 28(−5) and 36(−6) weeks post-surgery.

**Figure 6:**
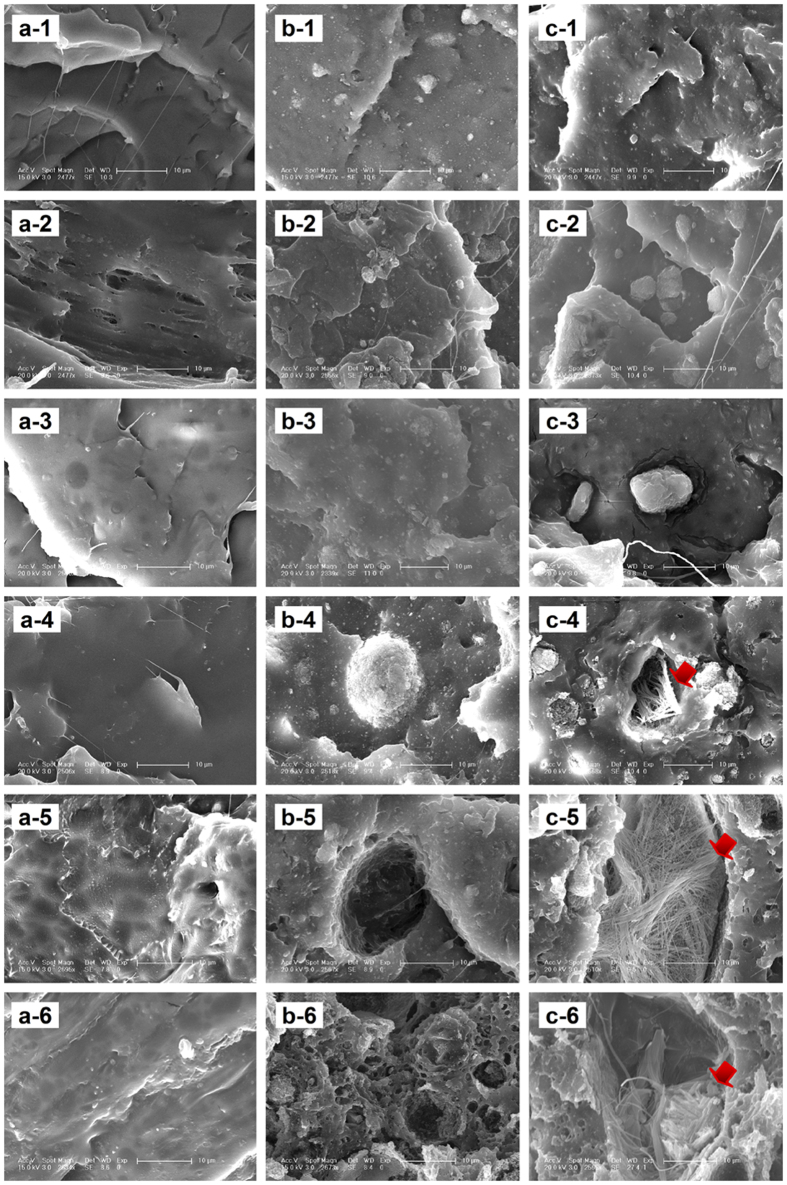
Fracture ESEM high-magnification micrographs of the intramuscular implants of PLLA (**a**), n-HAP/PLLA (**b**) and g-HAP/PLLA (**c**) at 0(−1), 4(−2), 12(−3), 20(−4), 28(−5) and 36(−6) weeks post-surgery.

**Figure 7:**
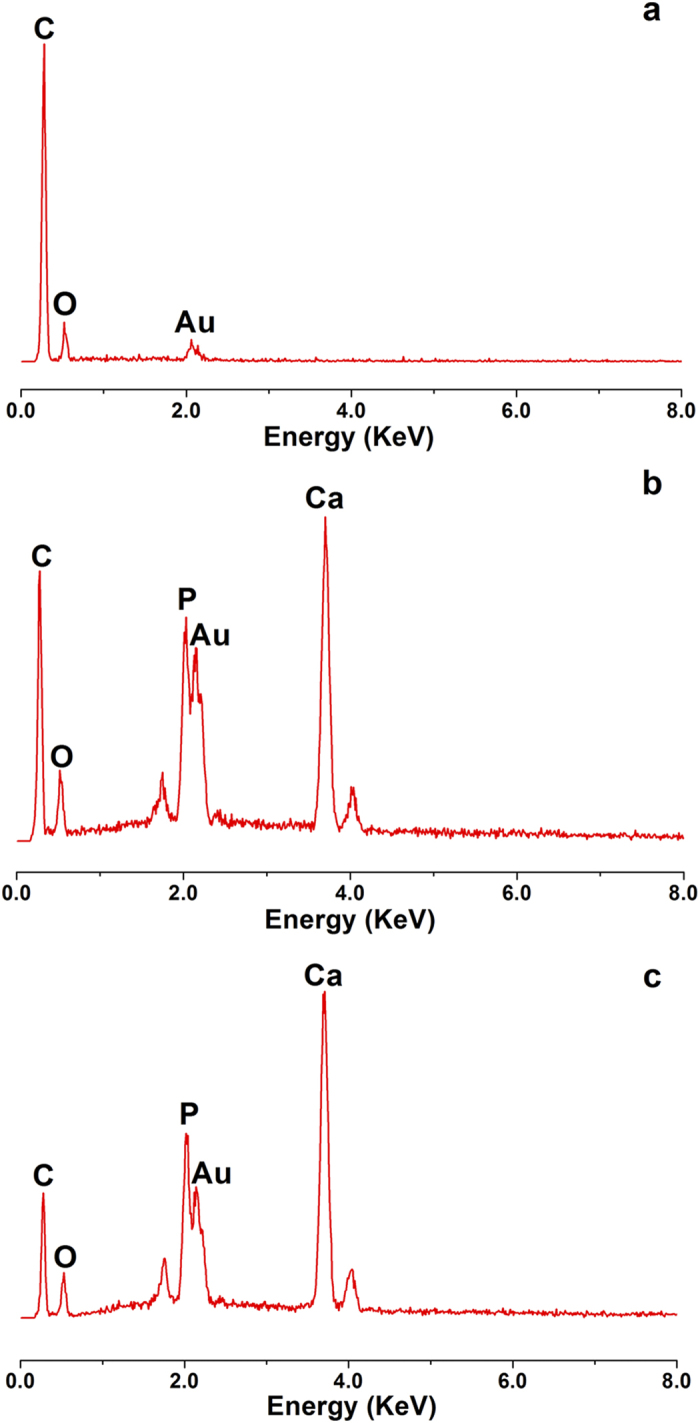
EDX analysis of the g-HAP/PLLA composites with red arrow shown in Fig. 6 at 20 (**a**), 28 (**b**) and 36 (**c**) weeks post-surgery, respectively.

The fracture ESEM micrographs of the samples were shown in Fig. 5. For pure PLLA samples, there were parallel fracture lines in the direction of stress (Fig. 5a) which might be due to the deformation of the matrix formed by external force. The fracture morphology of n- and g-HAP/PLLA composites was rougher than that of PLLA (Fig. 5b,c). It can be observed that n- and g-HAP particles diffused distribution in PLLA matrix. The n- and g-HAP particles significantly changed impact fracture morphology of PLLA matrix and large impact of the fault line were replaced by multiple fracture morphology. The morphological changes were far more marked for n- and g-HAP/PLLA composites than PLLA after comparable implantation times. With the *in vivo* degradation, n-HAP/PLLA composites showed an apparently porous surface morphology full of pores and hollows until 28 weeks (Fig. 5b1–5), and the porous surface morphology turned more obvious at 36 weeks (Fig. 5b–6). At 20 weeks, the fracture of g-HAP/PLLA composites showed visible cracks and wrinkles (Fig. 5c–4). Notable sags, gaps, and pores were apparent at 28 and 36 weeks (Fig. 5c5–6). However, no pore was detected with relative smooth structure on the PLLA fracture after 36 weeks of *in vivo* degradation (Fig. 5a2).As shown in Fig. 6, the microscopic changes were also clearly observed with ESEM under high-magnification. There was no obvious changes in pure PLLA materials at any time interval. While many pores were formed as increasingly disappeared of the HAP particles from the matrix over time. The pores appeared in g-HAP/PLLA composites was a little earlier than that of n-HAP/PLLA. Some sags and gaps were observed in g-HAP/PLLA composites at 12 weeks after implantation and then pores turned more obvious over time. While pores appeared only from 28 weeks after implantation in n-HAP/PLLA composites. As seen in Fig. 7, energy-dispersive X-ray spectrometry (EDX) analysis was evaluated on the pores formed in g-HAP/PLLA composites at 20, 28 and 36 weeks which showed in Fig. 6 with red arrows. The EDX analysis of area pointed by red arrow shown in Fig. 6c–4 indicated that the g-HAP particles disappeared from the pore and left the matrix. More interestingly, the EDX results of area pointed by red arrow shown in Fig. 6c–5,c-6 showed that fiber-like morphology was formed in the pores detecting with Ca and P elements.

## Discussion

An ideal absorbable device for bone fracture fixation should have a high initial strength, an appropriate modulus and retain strength as long as the healing fracture needs support^18^. In this paper, to improve the interface adhesion between PLLA and nanoparticle fillers and the mechanical properties of the PLLA based composites, we prepared g-HAP particles with grafting polymerization of L-lactide on the surface of n-HAP and g-HAP/PLLA composites as described in our previous studies^6^,^19^. The g-HAP particles could be more uniformly dispersed either in chloroform or in the PLLA matrix and showed improved adhesion with PLLA matrix. Consequently, the g-HAP/PLLA composites exhibited improved mechanical properties due to the reinforcing and toughening effects in the composites. That was because the grafted-PLLA molecules played a role of tie molecules between the fillers and the PLLA matrix. And the g-HAP particles played the role of the heterogeneous nucleating agents in the crystallization of the PLLA matrix. So the initial values of bending strength, modulus, impact strength and crystallinity of the g-HAP/PLLA composites were a little higher than that of n-HAP/PLLA composites or pure PLLA. High initial strength of the fixation device is necessary in order to cope with external and muscular loads after reduction of fracture. Even though the bending strength of g-HAP/PLLA composites prepared in the present study is lower than that produced by a forging process^5^ or by self-reinforced technique (SR-PLLA, BIONX, Finland), it will be suitable for the fixation of human non-load bearing bone fracture, such as cancellous bone fracture fixation^18^.

However, it is urgent to make clear that the influence of grafted HAP nanoparticles on the *in vivo* degradation behavior of composite implants as degradation rate is a critical factor affecting bone fracture healing. The degradation mechanism of biodegradable polymer is chemical degradation via hydrolysis and it was regarded that the chain ends cleavage resulted in mass loss, while random scission dominated the reduction in molecular weight^20^,^21^. So the uptake of water is considered to be specifically important for the degradation of the material. In our study, the molecular weight of the composites decreased faster from the early period than the pure PLLA. This is possibly because the body fluid could diffuse more easily into the composites than into pure PLLA as no chemical bonding existed between the particles and the PLLA matrix in the composites. Therefore, it’s deduced that the composites displayed a faster degradation than pure PLLA in well accordance with the literature^5^.

A general difficulty in composite science is the development of good adhesion between matrix and reinforcement. If the adhesion is insufficient the composite has poor strength and fatigue properties^22^. In this study, fluids can diffuse rapidly along the interface of n-HAP/PLLA composite due to poor adhesion and disrupt the interface which leads to rapid strength loss of the composite. It has been reported that the hydrolytic chain cleavage proceeded preferentially in the amorphous regions, and hence leading to the increase in polymer crystallinity^16^. In the present study, the g-HAP/PLLA composites demonstrated a significant faster decrease in molecular weight than n-HAP/PLLA. What’s more, ESEM results showed that the pores appeared in the g-HAP/PLLA composites were more rapidly than that of n-HAP/PLLA. This might be due to the dominated degradation occurred earlier on amorphous regions of grafted PLLA molecules on the HAP particles with uptake water from body fluid as the distribution and adhesion of g-HAP nanoparticles in the PLLA matrix was improved which has been shown in our previous study^19^. The well distributed of g-HAP nanoparticles helped in the easily invasion of body fluid into the inner of the g-HAP/PLLA composites from the interface between g-HAP nanoparticles and PLLA matrix. Subsequently, the degradation of amorphous regions of PLLA matrix in the g-HAP/PLLA composites occurred prior to the crystalline regions. As the polymer chains in amorphous regions degrade, the number of amorphous regions decrease, the proportion of crystalline to amorphous regions increased^15^,^23^, in agreement with the WAXD results (supplementary Figure 1). So the crystallinity of them increased gradually over time until 20 weeks after implantation. Afterwards, the crystalline region turned to be the dominant degradation region and resulted in the crystallinity decrease. Based on the above reasons, even if the crystallinity of n-HAP/PLLA composites increased over time until 20 weeks and the invasion of body fluid into the inner of the n-HAP/PLLA composites also occurred from the interface between n-HAP particles and PLLA matrix, the degradation of n-HAP/PLLA composites was a little slower than that of g-HAP/PLLA. However, water penetration into the pure PLLA samples was more difficult. So the degradation of PLLA was slower than that of the composites and its molecular weight decreased more slowly and the crystallinity increased until 28 weeks after implantation. The degradation of pure PLLA was also not observed apparently by ESEM.

An interesting and important question is what is necessary and secure strength retention time for absorbable fixation materials *in vivo*. The healing of cancellous bone fracture through trabecular bone growth is a much faster process (4–6 weeks) compared to the healing of cortical bone fractures (12–24 weeks)^24^. Corresponding to the faster decrease in molecular weight, the bending strength and modulus of the g-HAP/PLLA composites decreased faster than that of n-HAP/PLLA composites with *in vivo* degradation. The bending strength of g-HAP/PLLA composites maintained 51.0% of their initial values (58.14 MPa) at 20 weeks and 21.4% (24.40 MPa) at 36 weeks. This was higher than the strength of cancellous bone (10–20 MPa)^25^. While the impact strength increased obviously for the g-HAP/PLLA composites than that of n-HAP/PLLA. This might be due to small molecules produced by the firstly degradation of grafted PLLA molecules onto n-HAP particles played a role of plasticizer and hence improved the toughness of the composites. Small molecules produced by the slower degradation of PLLA matrix in n-HAP/PLLA composites also played a role of plasticizer and improved the toughness of the n-HAP/PLLA composites to some extent. But the increased of impact strength of the n-HAP/PLLA composites was lower than that of g-HAP/PLLA. The impact strength of g-HAP/PLLA composite decreased at 28 weeks post-surgery and exhibited an abrupt decline at 36 weeks post-surgery due to a faster *in vivo* degradation rate than n-HAP/PLLA composite. This results were in accordance with the change of tension-compression, molecular weight, Tg, Tm and ESEM morphology at 36 weeks post-surgery. In addition, the torsion test is also an important parameter of mechanical properties for bone fixation implants and it has been investigated with the PLA-based composites^26^. In this study, torsion test was also evaluated and the values of torsion firstly increased for the composites at 4 weeks because of absorbed body fluid and then decreased gradually with *in vivo* degradation.

With the *in vivo* degradation results, we can conclude that the interface between the g-HAP particles and PLLA matrix was more susceptible to erosion by the body fluid. Although this report are based on the mechanical, molecular weight and ESEM morphology data obtained from implants which were implanted in muscle tissue, we believe that these results are in agreement with the behavior of the same implants in bone tissue, because we found in several studies that the strength retention of absorbable rods is practically the same in subcutaneous tissue as in bone tissue^5^,^18^,^27^. According to the present study g-HAP/PLLA implants seem to be suitable in the treatment of cancellous bone fractures where the fixation needs high initial mechanical properties and fast degradation rate.

## Conclusions

The *in vivo* degradation of n- and g-HAP/PLLA composites were evaluated with mechanical properties, molecular weight, crystallinity, thermal behavior, and ESEM morphology. The g-HAP/PLLA composites showed the fastest degradation rate among all the materials and n-HAP/PLLA also exhibited faster degradation rate than pure PLLA in terms of molecular weight decrease, mechanical property changes and matrix erosion of micromorphology. This indicated that g-HAP/PLLA composite implants were more suitable for the bone fixation requiring rapid resorption. The results obtained from this *in vivo* study encourage the clinical use of the g-HAP/PLLA composites in the fixation of human non-load bearing bone fracture which needs high initial strength and fast degradation rate. Further long-term system studies for degradation of the g-HAP/PLLA materials are also needed.

## Methods

### Materials

PLLA with molecular weight 50,000 was prepared by the ring opening polymerization of the L-lactide in the presence of stannous octoate (Sn(Oct)_2_) as catalyst according to our previous study^6^. The preparation of hydroxyapatite nanoparticles (n-HAP) and the surface-grafted hydroxyapatite nanoparticles by PLLA (g-HAP) have been described in our previous papers^19^. In brief, n-HAP was synthesized according to the reaction shown in Equation 1:


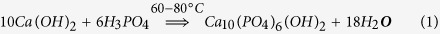


It was an acicular crystal of about 100 nm in length and 20–40 nm in width, with the atomic ratio Ca/P ≈ 1.67. Then, L-lactide was ring-opening polymerized onto the surface of n-HAP particles in the presence of stannous octoate (Sn(Oct)) as catalyst to obtain g-HAP according to Equation 2:


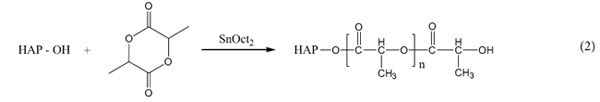


The amount of grafted polymer on the surface of g-HAP was determined by thermal gravimetric analysis to be about 5.0 wt%.

### Preparation of n-HAP/PLLA and g-HAP/PLLA composites

The n-HAP/PLLA and g-HAP/PLLA composites were prepared as follows. Pre-weighed dried n-HAP or g-HAP powders were uniformly suspended in 20 folds (in weight) chloroform with the help of magnetic stirring and ultrasonic treatment. And the suspension was added into a 10% (w/v) PLLA/chloroform solution to achieve the n- or g-HAP content of 10 wt% in the composite. The mixture was precipitated in an excess of ethanol, and the composite was dried in a vacuum-oven at 40–50 °C for 24 h to remove the residual solvent.

For preparing mechanical and *in vivo* test specimens, all composites were blended in Torque Rheometer at 190 °C for 5 min and laminated into sheets with a thickness of about 2 mm by hot press molding at 190 °C and 15 MPa, then the samples were annealed at 115 °C for 1 h. Rectangular bars having effective dimensions of 30 mm × 5 mm × 2 mm were cut from a 2-mm-thick plate. Unfilled PLLA materials (100% PLLA) were made by the same method as a control group.

### Intramuscular implantation

All the materials used in this study were sterilized with ethylene oxide at 55 °C for 4 h and implanted intramuscularly for *in vivo* degradation assessment. Rearing of the New Zealand White Rabbits and all experiments using them were carried out at the Institute of Surgery, China-Japan Union Hospital, Jilin University. The guidelines for animal experimentation of Jilin University were carefully observed in accordance with international standards on animal welfare and were approved by the Animal Research Committee of Jilin University. The rabbits were anesthetized by an intravenous injection of Nembutal (50 mg/kg body weight) and local administration of 0.5% (w/v) lidocaine. The operations were carried out under standard aseptic conditions. The implants were embedded into dorsal muscle of rabbits. Four parallel samples in a rabbit were used for each material. After surgery, the rabbits were kept in cages and maintained with a regular diet. All the rabbits were given a daily injection of penicillin for one week and were sacrificed by Nembutal overdose at 4, 12, 20, 28 and 36 weeks post-surgery. The implants were taken out and all samples before and after surgery were measured for mechanical properties, surface and fracture images, molecular weight and thermal properties.

### Mechanical properties

The bending strength of materials was measured by the three-point bending method using a universal testing machine (Instron 1121, UK) at a crosshead speed of 5 mm·min^−1^ with a span of 20 mm. The measurement of impact strength was performed on impact testing machine (JJ-20, China). The torsion test was performed on dynamic mechanical analyzer (MAK-04, Metravib, France).

### Surface and fracture morphology

The surface and impact fractures of PLLA, n-HAP/PLLA and g-HAP/PLLA composites were observed by an environmental scanning electron microscope (ESEM, XL30 FEG, Philips) connected to an energy-dispersive X-ray spectrometry (XL30W/TMP, Philips). X-ray intensities for calcium, phosphorus were analyzed across the fracture surfaces.

### Molecular weight change

The viscosity-average molecular weight (*M*v) of PLLA in the composites before and after implantation for 4, 12, 20, 28 and 36 weeks was determined from the intrinsic viscosity in chloroform at 25 °C using the following Equation 3:


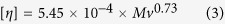


To remove the nanoparticles from the n-HAP/PLLA and g-HAP/PLLA composites, the samples were firstly dissolved in chloroform thoroughly and the solutions were centrifuged with a speed of 10,000 rpm. The supernatant was taken and repeated the centrifugation process for two times. Then ethanol was added into the supernatant for the PLLA sedimentation. Finally the PLLA was dried with vacuum and measured the intrinsic viscosity.

### Thermal properties and crystallinity measurement

The thermal properties of PLA, n-HAP/PLLA and g-HAP/PLLA composites were measured by the differential scanning calorimetry (DSC-7, Perkin-Elmer) at a heating rate of 10 °C·min^−1^ from 20 to 200 °C.

Crystallinity of the PLLA in the composites was calculated from the following Equation 4:


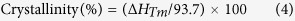


Where Δ*H*_Tm_ indicates the melting enthalpy (J/g) that was calculated from the fusion peak in DSC curve. And the value 93.7 (J/g) is the melting enthalpy of theoretical completely crystalline PLLA polymer (J/g)^28^.

### Statistical analysis

All quantitative datas were analyzed with Origin 8.0 (OriginLab Corporation, USA) and expressed as the mean ± standard deviation. Statistical comparisons were carried out using the analysis of variance (ANOVA One-way, Origin 8.0). A value of P < 0.05 was considered to be statistically significant.

## Additional Information

**How to cite this article**: Wang, Z. *et al.* A comparative study on the *in vivo* degradation of poly(L-lactide) based composite implants for bone fracture fixation. *Sci. Rep.* **6**, 20770; doi: 10.1038/srep20770 (2016).

## Supplementary Material

Supplementary Information

## Figures and Tables

**Table 1:** Statistical analysis of the data shown in Fig. 1a: change in bending strength of n- and g-HAP/PLLA and PLLA samples with time.

	Weeks after implantation					
	0	4	12	20	28	36
g-HAP/PLLA vs. n-HAP/PLLA	n.s	n.s	b	c	a	n.s.
g-HAP/PLLA vs. PLLA	n.s	n.s	c	c	c	c
n-HAP/PLLA vs. PLLA	n.s	n.s	c	d	c	c

aWith a significant difference at P < 0.02.

bWith a significant difference at P < 0.01.

cWith a significant difference at P < 0.005.

dWith a significant difference at P < 0.05.

en.s. = not significant.

**Table 2:** Statistical analysis of the data shown in Fig. 1b: change in bending modulus of n- and g-HAP/PLLA and PLLA samples with time.

	Weeks after implantation					
	0	4	12	20	28	36
g-HAP/PLLA vs. n-HAP/PLLA	n.s	n.s	n.s	c	n.s	n.s.
g-HAP/PLLA vs. PLLA	a	n.s	d	b	c	c
n-HAP/PLLA vs. PLLA	n.s	n.s	d	n.s	c	c

aWith a significant difference at P < 0.02.

bWith a significant difference at P < 0.01.

cWith a significant difference at P < 0.005.

dWith a significant difference at P < 0.05.

en.s. = not significant.

**Table 3:** Statistical analysis of the data shown in Fig. 1c: change in impact strength of n- and g-HAP/PLLA and PLLA samples with time.

	Weeks after implantation					
	0	4	12	20	28	36
g-HAP/PLLA vs. n-HAP/PLLA	n.s	n.s	n.s	c	b	c.
g-HAP/PLLA vs. PLLA	n.s	c	c	c	c	n.s
n-HAP/PLLA vs. PLLA	n.s	n.s	d	c	c	a

aWith a significant difference at P < 0.02.

bWith a significant difference at P < 0.01.

cWith a significant difference at P < 0.005.

dWith a significant difference at P < 0.05.

en.s. = not significant.

**Table 4:** Changes in crystallinity of PLLA, n- and g-HAP/PLLA after *in vivo* degradation at different time.

Time (weeks)	PLLA	n-HAP/PLLA	g-HAP/PLLA
0	19.5 ± 5.3	25.7 ± 5.1	22.1 ± 1.2
4	22.6 ± 6.2	25.6 ± 9.9	26.8 ± 7.5
12	24.4 ± 7.3	24.6 ± 7.6	29.7 ± 9.3
20	25.5 ± 4.4	29.7 ± 7.0	33.7 ± 8.7
28	27.1 ± 2.7	29.4 ± 8.7	33.3 ± 3.0
36	23.6 ± 4.2	25.5 ± 0.1	27.8 ± 8.0
