# Supplementary material for: A comparative study on the in vivo degradation of poly(L-lactide) based composite implants for bone fracture fixation
Source: Sci Rep. 2016 Feb 9;6:20770. doi: 10.1038/srep20770 (PMC4808906; doi:10.1038/srep20770)
Supplement: Supplementary Information [file srep20770-s1.doc]

**Supplementary information**

**A comparative study on the in vivo degradation of poly(L-lactide) based composite implants for bone fracture fixation**

Zongliang Wang ^a, d^, Yu Wang ^a^, [Yoshihiro Ito](http://www.sciencedirect.com/science/article/pii/S014296121400012X) ^b, c^, Peibiao Zhang ^a^* Xuesi Chen ^a^

^a^ *Key Laboratory of Polymer Ecomaterials, Changchun Institute of Applied Chemistry, Chinese Academy of Sciences, Changchun 130022, PR China*

^b^ *Nano Medical Engineering Laboratory, RIKEN, 2-1 Hirosawa, Wako, Saitama 351-0198 Japan*

^c^ *Emergent Bioengineering Materials Research Team, RIKEN Center for Emergent Matter Science, 2-1 Hirosawa, Wako, Saitama 351-0198, Japan*

^d^ *University of Chinese Academy of Sciences, Beijing 100039, PR China*


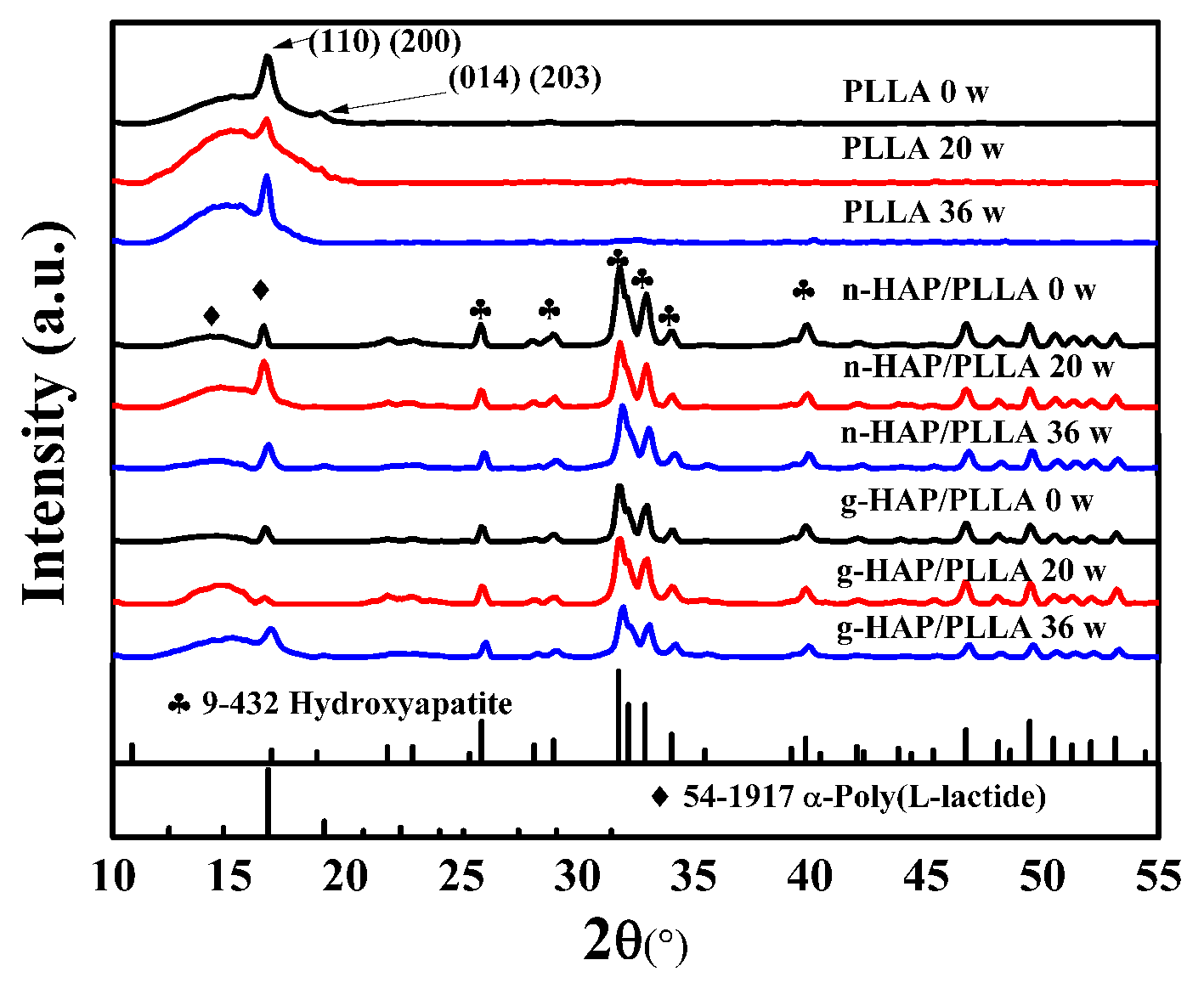


**Supplementary Figure 1.** WAXD patterns for PLLA, n-HAP/PLLA and g-HAP/PLLA 0, 20 and 36 weeks after implantation.
